# Evaluation of Safety of Iron-Fortified Soybean Sprouts, a Potential Component of Functional Food, in Rat

**DOI:** 10.1007/s11130-016-0535-8

**Published:** 2016-02-15

**Authors:** Małgorzata Kujawska, Małgorzata Ewertowska, Ewa Ignatowicz, Teresa Adamska, Hanna Szaefer, Magdalena Zielińska-Dawidziak, Dorota Piasecka-Kwiatkowska, Jadwiga Jodynis-Liebert

**Affiliations:** Department of Toxicology, Poznan University of Medical Sciences, 30 Dojazd Str., 60-631 Poznań, Poland; Department of Pharmaceutical Biochemistry, Poznan University of Medical Sciences, 4 Święcicki Str., 60-781 Poznań, Poland; Department of Food Biochemistry and Analysis, Poznan University of Life Sciences, 48 Mazowiecka Str., 60-623 Poznań, Poland

**Keywords:** Ferritin, Soybean sprouts, Clinical chemistry, Haematology, Antioxidant enzymes

## Abstract

Ferritin-iron is currently considered as one of the most promising iron forms to prevent iron deficiency anaemia. We found that the cultivation of soybean seeds in a solution of ferrous sulfate results in material with extremely high iron content - 560.6 mg Fe/100 g of dry matter, while ferritin iron content was 420.5 mg/100 g dry matter. To assess the potential adverse effects of a preparation containing such a high concentration of iron, male and female Wistar rats were exposed via diet to 10, 30, 60 g soybean sprouts powder/kg feed for 90 days. There were no differences in final body weight and mean food consumption between controls and rats administered sprouts. No statistically significant differences in haematology and clinical chemistry parameters were found between controls and treated rats. Microscopic examination of 22 tissues did not reveal any pathology due to soybean sprouts intake. Long term administration of the test material did not cause oxidative damage to DNA and protein in the liver as evidenced by the unchanged basal levels of DNA damage as well as carbonyl groups content. Lipid peroxidation was slightly increased only in females. The activity of several antioxidant enzymes: superoxide dismutase, glutathione peroxidase and glutathione S-transferase was increased, which substantially enhanced the antioxidant status in the liver from the rats treated with soybean sprouts. Hence, the material tested can be recommended as a component of food supplements for individuals with iron deficiency anaemia and inflammatory bowel diseases.

**Electronic supplementary material:**

The online version of this article (doi:10.1007/s11130-016-0535-8) contains supplementary material, which is available to authorized users.

## Introduction

Iron deficiency anaemia is the most common form of malnutrition in the world. Unfortunately, an increase in inorganic iron supply to the human organism does not provide expected results due to many side effects and poor absorption from the digestive tract. Ferritin-iron is currently considered as one of the promising iron forms to prevent iron deficiency anaemia and to treat inflammatory bowel diseases [1] Plant ferritin is a protein which complexes iron atoms in the apo-protein shell and is expressed in all plant cells in order to protect them from toxic effect of this element [2]*.* The highest concentration of ferritin is noted in non-green plastids of leaves, roots and seeds amyloplasts [3, 4]. The content of naturally occurring ferritin in edible parts of plants differs greatly and its high content is noted especially in legume seeds. It reaches the level of 50–70 mg/kg and corresponds to ~10 mg of iron per kg [5]*.* Thus, the amount of iron is still insufficient to apply these seeds as an iron supplement, even though high absorption of ferritin iron has been documented in clinical and animal studies. But the concentration of ferritin and ferritin iron may be increased with biofortification of plants, both with genetic engineering and with modification of cultivation conditions [6]. Extremely high content of iron in plants has been obtained in legume seeds sprouting in the FeSO_4_ solutions. In the sprouts, most of the accumulated iron was complexed with ferritin, but of course Fe(II) ions were also present in this material. The prepared soybean sprouts, with almost 70-fold increased iron content, have been proposed as a supplement of this element for iron-deficient rats and it has been proven that the preparation was a source of well absorbed iron [7, 8]. Ferric sulfate has been accepted both as a food ingredient (used as a supplement and food fortificant) and as a component of edible plant fertilizers [7]. Therefore, it could be expected that the preparation should be absolutely safe for the consumer. However, the introduction of a new source of iron to the human diet always raises serious concerns about the iron toxicity, especially related to oxygen stress induction. Dietetic preparation with such a high iron concentration may cause undesired effects in the organism after oral administration. Hence, the aim of the present study was to investigate the safety of the dietary administration of soybean sprouts containing very high concentration of iron, to rats for 90 days.

## Materials and Methods

### Test Material

#### Sprouts Preparation

Soybean seeds (*Glycine max*, Aldana var.), obtained from the Department of Genetics and Plant Breeding, Poznań University of Life Sciences were used. The seeds were cultured in a special climate chamber (ADAPTIS, Conviron) on germination trays for seven days at room temperature under controlled illumination. They were watered every day with fresh 20 mM FeSO_4_ solution. Then the sprouts were dried in a stream of air till 8–10 % of moisture was obtained. The temperature gradient applied on drying (80–40 °C) was chosen to provide the optimal microbial purity of the preparation. Samples of dried sprouts were milled and stored as a powder in tightly closed containers at room temperature. Soybean seeds were cultured without FeSO_4_ in the same conditions. All listed below chemical analyses were performed concurrently in both kinds of sprouts.

#### Iron Content Determination

Total iron content was analysed by atomic absorption spectroscopy (λ = 248.3, gaps width of 0,15 nm) after mineralization of sprouts. The inorganic iron content, not chelated and not introduced into organic compounds was determined spectrophotometrically (λ = 470 nm) using thiocyanate reaction in the environment of hydrochloric acid (pH < 2). The difference between total and inorganic iron content gives the complexed form of iron, which is assumed to be the ferritin iron content [9]*.*

#### Total Antioxidant Capacity

Total antioxidant potential of the sprouts was determined by the total peroxyl radical-trapping potential (TRAP) method in methanolic extracts. The time necessary for oxidation of DCFH-DA probe (2,7-dichlorodihydrofluorescein diacetate) to highly fluorescent dichlorofluorescein (DCF) by the radicals generated from AAPH (2,2′-azobis(2-amidinopropane)-dihydrochloride) in the presence of methanolic extracts was measured. The excitation and emission wavelength were 480 and 520 nm, respectively. Calibration curve was prepared in the range of 0–100 mg Trolox (y = 22.642× + 298.81, R^2^ = 0.9968). TRAP value was expressed as milimoles of Trolox equivalents (TrxE)/kg dry plant material [10].

#### Total Phenolic and Total Flavonoid Compounds Determination

The total phenolic compounds content (TPC) in methanolic extract was determined by the Folin–Ciocalteu method [11]. Gallic acid (0–0.7 μg/mL) was used to produce a standard calibration curve (y = 0.0109×, R^2^ = 0.9959). The total phenolic content was expressed in mg of gallic acid equivalents (GAE)/g dry plant material. Total flavonoid content (TFC) was measured by aluminium chloride colorimetric assay using the method based on the formation of complex flavonoid-aluminium [11]. Quercetin (0–30 μg/mL) was used to produce a standard calibration curve (y = 0.0396×, R^2^ = 0.9897). The total flavonoid content was expressed in mg of quercetin equivalents (QE)/g dry plant material.

### Statistical Analysis

All analyses were performed in seven replicates and the results were expressed as the average value ± standard deviation.

### *In vivo* Experiment

#### Chemicals

The reagent kit for protein carbonyl determination was purchased from BioCell Corp. Ltd. (New Zealand). Agarose for comet assay was purchased from Prona (USA). Other chemicals were from Sigma-Aldrich (St Louis, USA) and local suppliers.

#### Animals and Dose Formulations

Wistar rats bred in the Department of Toxicology, Poznań University of Medical Sciences, Poland, were used. Animals were held (four rats/cage) in polycarbonate cages (Techniplast, Italy) with wood shavings in a room maintained under 12 h light/dark cycle, 21 ± 2 °C, 40–54 % relative humidity. Food and drinking water were available *ad libitum.* Soybean sprouts powder was mixed into certified laboratory feed Labofeed H (ISO 22 000) to get concentration 10, 30, 60 g/kg feed and pellets were prepared.

#### Experimental Design

Forty male and forty female Wistar rats weighing 215 ± 22 g and 158 ± 13 g, respectively, were divided randomly into four groups: controls and exposed *via* diet to 10, 30, 60 g of sprouts/kg feed for 90 consecutive days. Control animals were given feed without any additives. Animals were observed twice a week for signs of toxicity. Body weight was recorded weekly during the study. Feed consumption was assessed by weighing the feeders. The intake of test substance was calculated weekly from the nominal test substance concentration, the feed consumption and mean body weight. At 91 day of experiment the rats were anesthetized with ketamine, blood was withdrawn from the heart and the organs were harvested for histopathological examinations. The livers were perfused with ice cold 1.15 % KCl and their portions were dissected and stored at -80 °C for determination of biochemical parameters.

The experiment was performed according to the guidelines of the Local Ethics Committee for Animal Experimentation.

#### Clinical Pathology

The following haematological parameters were evaluated: erythrocyte count (RBC), total (WBC) and differential leukocyte count (neutrophils, lymphocytes, monocytes, eosinophils and basophils), haemoglobin (HGB), and platelet count (PLT). Haematological parameters were determined using an automated haematology analyser Cell-Dyn 3700 (Abbott Laboratories, Illinois, USA). Clinical chemistry parameters evaluated in separated serum included alanine aminotransferase, aspartate aminotransferase, alkaline phosphatase, total cholesterol, total protein, glucose, blood urea nitrogen, creatinine, calcium, sodium, potassium, inorganic phosphorus, and chloride. Serum clinical chemistry parameters were determined using a chemistry analyser XL 300 (Erba Diagnostics, Mannheim, Germany) and reagents purchased from Biosystems (Spain).

#### Anatomic Pathology

At the time of necropsy the rats were examined grossly and the following organs were collected: adrenals, aorta, bone marrow, brain, colon, oesophagus, heart, ileum, kidneys, liver, lungs, ovaries, pancreas, pituitary gland, spleen, stomach, testes, thyroid, trachea, urinary bladder, and uterus. The tissues were fixed in 4 % buffered formalin solution for 24 h and processed for paraffin embedding by standard methods. The paraffin blocks were sectioned at 5 μm, stained with haematoxylin and eosin and examined by light microscopy.

#### Biochemical Assays

The level of lipid peroxidation in the liver homogenate was assessed by measuring thiobarbituric acid reactive substances (TBARS) [12]. Reduced glutathione content in the liver was assayed with Ellman’s reagent [13]. Protein carbonyl concentration in the liver was determined by the ELISA method according to the manufacturer instruction. Alkaline comet assay in the liver was performed according to the method described by Hartman and others [14]. Antioxidant enzymes were assayed in the liver cytosol. The superoxide dismutase (SOD) assay was based on inhibition of spontaneous epinephrine oxidation [15]. Catalase (CAT) activity was determined by the measurement of hydrogen peroxide reduction [15]. Glutathione peroxidase (GPx) activity was determined using hydrogen peroxide as a substrate; the disappearance of NADPH was a measure of the enzyme activity [16]. Glutathione reductase (GR) activity was assayed by measuring NADPH oxidation using as a substrate oxidized glutathione [16].

### Statistical Analysis

The GraphPad InStat statistical package (version 3) was used. The mean values and standard deviations were calculated separately for males and females. One way analysis of variance (ANOVA) followed by the Student-Newman-Keuls test for multiple comparisons was used. *p* < 0.05 was considered the limit of significance.

## Results and Discussion

The difference between the heme-iron and ionic iron systems of the plant ferritin absorption suggests that the protein may be an abundant source of this element for individuals with disturbed uptake mechanisms [17]. Legume seeds are the richest in ferritin among edible parts of plants. We found that the cultivation of soybean seeds in a solution of FeSO_4_ permits obtaining material with extremely high iron content of 560.6 mg/100 g of dry matter, while the ferritin iron content was ~420.5 mg/100 g d.m. It means that the ferritin iron represented ~75 % of total iron in the obtained preparation. In sprouts cultured in distilled water the total iron or ferritin iron content was distinctly lower: 11.0 mg/100 g d.m. and 1.8 mg/100 g d.m., respectively. Moreover, the powdered soybean sprouts have been found not only a source of iron, but also various bioactive compounds. Abiotic stress influence reactive oxygen species formation, both as a consequence of hydrolytic processes and oxidative conditions. Apart from ferritin other compounds with strong antioxidant properties are formed while sprouting in the described conditions [18]. TRAP determined in the material tested was 5.57 ± 0.16 mM TrxE/kg dry matter and this value corresponds to the relatively high concentration of both total phenolic and flavonoid compounds: 3.55 ± 0.02 mg GAE/g d.m. and 0.185 ± 0.02 mg QE/g d.m., respectively. The TRAP value for sprouts cultured in water was slightly higher, 7.16 ± 2.21, whereas the concentration of total phenolic or flavonoid compounds was similar to that found in iron-fortified sprouts: 4.01 ± 0.03 mg GAE/g d.m. and 0.222 ± 0.06 mg QE/g d.m., respectively. Thus, it could be expected that potential pro-oxidant effects associated to the high content of iron would be cancelled by the presence of antioxidant compounds in the material tested. To assess the potential adverse effects of the preparation containing such a high concentration of iron, soybean sprouts powder was administered to rats for 90 days via diet. On the basis of food consumption measurements and the nominal dietary concentration of the material tested, the calculated mean daily intake of soybean sprouts was 0.63, 1.96, 3.90 g/kg body weight/day in males and 0.50, 1.25, 4.10 g/kg body weight/day in females. There were no statistically significant differences in final body weight and mean food consumption between controls and rats administered sprouts (data not shown). No statistically significant differences in haematology and clinical chemistry parameters were found between controls and treated rats (Tables I and II, supplementary material). Microscopic examination of 22 tissues did not reveal any pathology due to soybean sprouts intake (data not shown). A fundamental role of iron in generation of reactive oxygen species, prompted us to investigate the markers of oxidative damage to hepatic lipids, protein and DNA. No changes in the levels of carbonyl groups (marker of protein oxidation) as well as DNA damage (determined by comet assay) were found. The level of lipid peroxidation, expressed as TBARS concentration, was slightly elevated, by 22–28 %, only in females. This increase was statistically significant in mid-dose and high-dose group (Table 1). Our results showed that the basal level of TBARS was greater in females than in males which has been noted also by other authors [19, 20]. Hence, it could be hypothesized that the slightly weaker antioxidant status of females rendered them more susceptible to ferric ions ingested with soybean sprouts. However, it cannot be translated to humans since there are no data concerning the sex-depended differences in antioxidant status. No substantial difference in the content of reduced glutathione (GSH), an essential cell antioxidant, between controls and treated animals was observed (Table 1). The results of the antioxidant enzymes activity determination are depicted in Table 2. The most consistent changes were observed for two glutathione related enzymes, glutathione peroxidase (GPx) and glutathione reductase (GR). GPx activity was increased in all (except for high-dose males) groups by 13–58 %. In all groups of rats a decrease in GR activity by 22–34 % was found. These changes, although consistent, were not dose related. The activity of superoxide dismutase (SOD) was increased in all female groups by 27–54 % and in one low-dose male group. Changes in catalase activity were diversified: 19 % decrease in low-dose male and female group, 20 % increase in mid-dose females and no changes in high-dose groups. Similarly, S-glutathione transferase activity was changed only in some animals, showing 32 and 53 % increase in mid- and high-dose female groups, respectively. A decrease in GR activity observed in all treated groups of rats can be ascribed to the inhibition of the enzyme by some flavonoids, components of soybean sprouts. Also other authors have reported a decrease in the activity of GR in the liver of rats treated with quercetin and genistein [21] or in rat liver cells incubated with delphinidin, (−)epicatechin, kaempferol, quercetin, luteolin, naringenin and apigenin [22].

**Table 1:** Effect of soybean sprouts on oxidative stress parameters in the liver of rats

Parameter	Sex	Dietary soybean sprouts (g/kg feed)			
		0	10	30	60
Lipid peroxidation	M	16.0 ± 1.4	15.4 ± 2.3	17.0 ± 1.8	15.5 ± 2.0
(nmol TBARS/mg protein)	F	25.7 ± 2.8	30.5 ± 3.8	31.4 ± 3.9* [↑22 %]	32.8 ± 4.1** [↑28 %]
Carbonyl groups	M	0.137 ± 0.150	0.134 ± 0.021	1.140 ± 0.026	0.107 ± 0.031
(nmol/mg protein)	F	0.111 ± 0.024	0.133 ± 0.024	0.104 ± 0.027	0.117 ± 0.034
DNA damage	M	68 ± 10	69 ± 4	70 ± 8	66 ± 8
(arbitrary points)	F	76 ± 4	73 ± 7	75 ± 6	73 ± 7
Reduced glutathione	M	2.49 ± 0.09	2.46 ± 0.33	2.41 ± 0.21	2.64 ± 0.14
(μmol/g liver)	F	2.03 ± 0.21	2.29 ± 0.10* [↑13 %]	2.20 ± 0.12	2.24 ± 0.27

TBARS – thiobarbituric acid reactive substances

*Significantly different from controls

Values in parentheses express percentage of change

**Table 2:** Effect of soybean sprouts on antioxidant enzymes activity in the liver of rats

Parameter	Sex	Dietary soybean sprouts (g/kg feed)			
		0	10	30	60
Superoxide dismutase (SOD)	M	27.1 ± 2.8	31.9 ± 3.0* [↑18 %]	27.0 ± 3.5	26.3 ± 3.5
(U/mg protein)	F	20.8 ± 1.8	29.3 ± 4.6* [↑41 %]	32.1 ± 3.5* [↑54 %]	26.4 ± 4.8* [↑27 %]
Catalase (CAT)	M	5.07 ± 0.55	4.11 ± 0.44* [↓19 %]	4.87 ± 0.62	4.76 ± 0.59
(U/mg protein)	F	3.32 ± 0.33	2.69 ± 0.35* [↓19 %]	3.99 ± 0.49* [↑20 %]	3.59 ± 0.49
Glutathione peroxidase (GPx)	M	774.1 ± 64.2	873.6 ± 60.9* [↑13 %]	970.3 ± 90.2* [↑25 %]	787.6 ± 71.3
(nmol NADPH/min/mg protein)	F	768.9 ± 88.3	1121.2 ± 144.3* [↑46 %]	1213.5 ± 69.7* [↑58 %]	998.1 ± 50.4* [↑30 %]
Glutathione reductase (GR)	M	57.0 ± 6.3	40.5 ± 2.7* [↓29 %]	44.6 ± 4.8* [↓22 %]	41.5 ± 0.8* [↓27 %]
(nmol NADPH/min/mg protein)	F	49.4 ± 6.1	32.6 ± 4.9* [↓34 %]	36.7 ± 4.7* [↓26 %]	35.4 ± 4.1* [↓28 %]
Glutathione S-transferase (GST)	M	607.9 ± 57.5	652.7 ± 66.6	623.7 ± 75.8	661.0 ± 55.7
(nmol CDNB/min/mg protein)	F	390.0 ± 32.3	440.9 ± 69.7	514.2 ± 62.9* [↑32 %]	595.7 ± 55.3* [↑53 %]

Means and standard deviations are presented. *n* = 8

*Significantly different from controls

Values in parentheses express percentage of change

The results of our experiment demonstrated that soybean sprouts fortified with iron did not induce any changes in haematological and clinical chemistry parameters, and in organs morphology. As the presence of poorly complexed iron cannot be ruled out in *in vivo* conditions, we examined also the effect of long term administration of the material tested on the main cellular macromolecules in liver and found that no damage to proteins and DNA occurred, and lipid peroxidation level was slightly increased only in females. The activity of several antioxidant enzymes was increased, which substantially enhanced antioxidant status in the liver of rats treated with soybean sprouts. Hence, the material tested might be recommended as a component of food supplements for individuals with iron deficiency anaemia and inflammatory bowel diseases. It can be suggested that 1 g of iron-fortified sprouts covers 25 or 60 % RDA established for healthy adult woman or man, respectively [23].

## Electronic supplementary material

Supplementary Material Table I (DOC 38 kb)

Supplementary Material Table II (DOC 50 kb)
