# Supplementary material for: Evaluation of Safety of Iron-Fortified Soybean Sprouts, a Potential Component of Functional Food, in Rat
Source: Plant Foods Hum Nutr. 2016 Feb 15;71:13–8. doi: 10.1007/s11130-016-0535-8 (PMC4786607; doi:10.1007/s11130-016-0535-8)
Supplement: Supplementary file 1 — Table I (DOC 38 kb) [file 11130_2016_535_MOESM1_ESM.doc]

**Table I. Hematology findings in rats exposed to soybean sprouts.**

| PARAMETER | SEX | DIETARY SOYBEAN SPROUTS (g/kg feed) | | | |
| --- | --- | --- | --- | --- | --- |
|  |  | 0 | 10 | 30 | 60 |
| White blood cell count (G/l) | M | 5.1±1.3 | 5.3±2.6 | 5.2±1.7 | 4.9±1.58 |
|  | F | 1.9±1.0 | 3.1±1.2 | 2.8±0.6 | 2.3±1.4 |
| Neutrophils (%) | M | 6.5±1.7 | 7.4±2.4 | 7.9±2.7 | 8.6±1.8 |
|  | F | 5.6±2.9 | 4.3±1.5 | 5.3±2.5 | 6.4±1.8 |
| Lymphocytes (%) | M | 88.4±2.9 | 88.6±2.7 | 86.2±2.9 | 84.1±2.6 |
|  | F | 89.8±3.8 | 92.5±1.8 | 91.0±2.6 | 88.3±2.7 |
| Monocytes (%) | M | 3.9±0.8 | 3.3±0.7 | 4.4±1.1 | 5.7±1.6 |
|  | F | 3.3±1.3 | 2.2±0.5 | 2.6±0.6 | 3.9±1.1 |
| Eosinophils (%) | M | 0.9±0.6 | 0.8±0.3 | 1.2±0.6 | 1.3±0.5 |
|  | F | 1.1±0.5 | 0.7±0.3 | 0.8±0.4 | 1.1±0.5 |
| Basophils (%) | M | 0.4±0.3 | 0.4±0.3 | 0.2±0.2 | 0.4±0.3 |
|  | F | 0.3±0.2 | 0.3±0.1 | 0.27±0.1 | 0.4±0.3 |
| Erythrocyte count (T/l) | M | 8.9±0.4 | 8.7±0.3 | 8.6±0.3 | 9.1±0.3 |
|  | F | 8.5±0.5 | 8.0±0.3 | 8.0±0.3 | 8.0±0.2 |
| Hemoglobin (g/l) | M | 148.4±2.7 | 146.8±3.9 | 145.5±5.9 | 151.8±4.4 |
|  | F | 147.9±7.2 | 144.4±2.6 | 143.0±6.0 | 141.8±6.0 |
| Platelet count (G/l) | M | 1363.1±102.4 | 1275.0±61.7 | 1222.1±102.3 | 1394.0±100.3 |
|  | F | 1263.1±124.9 | 1261.4±86.5 | 1230.5±129.7 | 1211.9±67.8 |

Means and standard deviations are presented. n = 8
