# Supplementary material for: Evaluation of Safety of Iron-Fortified Soybean Sprouts, a Potential Component of Functional Food, in Rat
Source: Plant Foods Hum Nutr. 2016 Feb 15;71:13–8. doi: 10.1007/s11130-016-0535-8 (PMC4786607; doi:10.1007/s11130-016-0535-8)
Supplement: Supplementary file 2 — Table II (DOC 50 kb) [file 11130_2016_535_MOESM2_ESM.doc]

**Table II.** Clinical chemistry findings in rats exposed to soybean sprouts.

| PARAMETER | SEX | DIETARY SOYBEAN SPROUTS (g/kg feed) | | | |
| --- | --- | --- | --- | --- | --- |
|  |  | 0 | 10 | 30 | 60 |
| Alanine aminotransferase (U/l) | M | 50.9±6.4 | 43.0±6.4 | 44.9±11.4 | 45.2±5.2 |
|  | F | 49.0±15.7 | 35.3±10.2 | 34.6±9.2 | 38.9±10.6 |
| Aspartate aminotransferase (U/l) | M | 129.1±20.0 | 116.0±20.7 | 121.5±21.1 | 129.1±24.8 |
|  | F | 131.7±45.0 | 138.1±25.0 | 161.6±32.8 | 162.3±28.5 |
| Alkaline phosphatase (U/l) | M | 89.5±17.1 | 85.5±18.0 | 91.6±25.9 | 93.1± 10.8 |
|  | F | 83.9±19.4 | 82.3±30.9 | 77.0±22.5 | 56.5±10.7 |
| Total protein (g/l) | M | 64.2±2.4 | 62.4±1.5 | 60.3±2.4 | 65.1±1.4 |
|  | F | 65.3±2.8 | 61.1±3.8 | 61.1±4.1 | 58.9±3.4 |
| Total cholesterol (mmol/l) | M | 1.8±0.3 | 1.7±0.2 | 1.6±0.1 | 1.8±0.2 |
|  | F | 2.2±0.2 | 1.8±0.3 | 1.8±0.3 | 1.8±0.4 |
| Chloride (mmol/l) | M | 92.4±5.6 | 94.7±1.6 | 83.7±10.2 | 94.1±4.4 |
|  | F | 95.5±4.02 | 91.8±2.6 | 91.6±1.8 | 91.6±2.4 |
| Inorganic phosphorus (mmol/l) | M | 2.7±0.2 | 2.68±0.4 | 2.6±0.2 | 2.9±0.5 |
|  | F | 2.93±0.7 | 2.44±0.3 | 2.7±0.2 | 2.5±0.3 |
| Glucose (mmol/l) | M | 10.5±2.5 | 9.4±1.9 | 8.6±1.0 | 9.1±1.8 |
|  | F | 7.1±1.8 | 7.7±0.8 | 7.7±1.3 | 7.6±1.1 |
| Creatinin (mmol/l) | M | 48.9±9.3 | 49.8±3.3 | 59.6±3.7 | 60.9±4.5 |
|  | F | 57.1±6.4 | 45.7±12.0 | 56.1±10.5 | 63.9±8.7 |
| Blood urea (mmol/l) | M | 8.37±1.0 | 8.5±1.2 | 8.0±1.1 | 8.9±1.1 |
|  | F | 8.9±2.9 | 8.63±1.3 | 7.8±1.2 | 8.3±1.2 |
| Potassium (mmol/l) | M | 4.8±0.5 | 4.7±0.4 | 4.7±0.3 | 5.1±0.8 |
|  | F | 6.39±2.9 | 5.0±1.0 | 4.9±0.4 | 4.9±0.4 |
| Sodium (mmol/l) | M | 137.86±2.1 | 139.2±1.5 | 141.8±1.6 | 140.1±1.8 |
|  | F | 139.05±2.4 | 139.2±1.2 | 141.6±1.8 | 140.8±2.3 |
| Calcium (mmol/l) | M | 2.6±0.1 | 3.1±0.1 | 2.5±0.1 | 2.6±0.2 |
|  | F | 2.6±0.2 | 2.9±0.2 | 2.5±0.1 | 2.5±0.1 |

Means and standard deviations are presented. n = 8
